# Clinical Feasibility of AI‐Driven Automated Virtual Dental Implant Placement: A Cross‐Sectional Comparative Study

**DOI:** 10.1111/cid.70111

**Published:** 2025-12-22

**Authors:** Bahaaeldeen M. Elgarba, Rocharles Cavalcante Fontenele, Eslam Abdelwahab Dawood, Pierre Lahoud, Jan Meeus, Reinhilde Jacobs

**Affiliations:** ^1^ OMFS‐IMPATH Research Group, Department of Imaging and Pathology, Faculty of Medicine KU Leuven Leuven Belgium; ^2^ Department of Prosthodontics, Faculty of Dentistry Tanta University Tanta Egypt; ^3^ Department of Oral and Maxillofacial Surgery University Hospitals Leuven Belgium; ^4^ Department of Stomatology, Public Health and Forensic Dentistry, Division of Oral Radiology, School of Dentistry of Ribeirão Preto University of São Paulo (USP) Ribeirão Preto Brazil; ^5^ Division of Periodontology and Oral Microbiology, Department of Oral Health Sciences KU Leuven Leuven Belgium; ^6^ Department of Conservative Dentistry, Periodontology and Digital Dentistry LMU University Hospital, LMU Munich Munich Germany; ^7^ Department of Dental Medicine Karolinska Institute Stockholm Sweden

**Keywords:** 3D imaging, artificial intelligence, cone‐beam computed tomography, dental implant, implant dentistry

## Abstract

**Objectives:**

To assess the clinical validity of artificial intelligence (AI)‐driven virtual implant placement compared to human intelligence (HI)‐based virtual and actual single implant placement in the posterior mandible.

**Material and Methods:**

Thirty‐two patients for whom experts performed single implant placement in the posterior mandible were included, each with preoperative and postoperative cone‐beam computed tomography (CBCT) scans. The preoperative scans were registered to the corresponding postoperative scans and used for both AI‐ and HI‐driven virtual implant planning at the implant site. From each case, three implants' scenarios (i.e., HI‐placed, AI‐planned, and HI‐planned) were exported and compared. The analysis focused on angular deviation and the spatial relationship of each implant to adjacent anatomical structures and the expert‐designed prosthetic wax‐up. In addition, pairwise comparisons were performed to quantify angular and linear deviations at both the coronal and apical levels. Implant length and diameter from planned versus placed implants were evaluated, and planning time and consistency were compared between AI‐ and HI‐based approaches.

**Results:**

AI‐based planning showed no statistically significant differences compared to HI‐based methods observed in angular deviation relative to adjacent tooth (HI‐placed: 7.7° ± 5.6°, AI: 6° ± 4.7°, HI‐planned: 5.2° ± 5.7°) and coronal deviation (AI vs. HI‐placed: 0.9 ± 0.8 mm, AI vs. HI‐planned: 0.8 ± 0.4 mm, HI‐planned vs. HI‐placed: 1.0 ± 1.1 mm), all with *p* > 0.05. Implant diameter and length were also consistent across the different approaches, with HI‐placed (4.3 ± 0.3 mm; 9.7 ± 1.3 mm), AI (4.3 ± 0.4 mm; 9.9 ± 1.2 mm), and HI‐planned (4.3 ± 0.4 mm; 9.8 ± 1.3 mm) showing no significant differences (*p* > 0.05). However, AI planning was significantly faster (36.3 ± 7.3 s vs. 373 ± 113 s) and more consistent, with a median surface deviation of 0 mm compared to 0.39 mm for HI (*p* < 0.05).

**Conclusion:**

The AI tool showed clinically valid implant selection, matched expert placement and planning in virtual implant positioning for missing mandibular premolars and molars while being highly consistent and 10 times faster compared to human expert planning.

## 1 Introduction

The clinical success of dental implants is largely dependent on effective preoperative planning. The introduction of three‐dimensional (3D) imaging modalities, such as cone‐beam computed tomography (CBCT) and intraoral scanning (IOS), has greatly enhanced clinicians' ability to assess each patient's anatomical characteristics [1, 2, 3, 4]. Moreover, advances in digital technologies that process these imaging data have made implant planning more precise and clinically relevant [1]. These advances allow accurate translation of virtual plans into the clinical setting using computer‐aided design and computer‐aided manufacturing (CAD/CAM) to manufacture surgical guides for fully or partially guided dental implant placement [5, 6].

With the rapid advancement of these dental technologies, clinicians are often challenged to keep pace with evolving digital workflows. Mastery of these technologies typically requires substantial time and training, and even then, their integration into daily clinical practice can be time‐consuming [7, 8, 9]. Additionally, human variability in implant planning, due to differences in clinical background and experience, introduces potential biases that may affect the consistency and reproducibility of treatment outcomes [7, 10, 11, 12]. Consequently, artificial intelligence (AI) has been introduced to streamline various steps of the preoperative planning process to reduce clinician workload. By automating complex tasks, AI can significantly enhance time efficiency, allowing clinicians to focus afterwards on reviewing, approving, or modifying the proposed implant planning [7, 13, 14].

In the digital implant workflow, AI has been validated for a number of preoperative planning tasks. These include segmentation of jaws, teeth, mandibular canal, maxillary sinuses, implant restorations, and prosthetic crowns, as well as the registration of CBCT and IOS data across various clinical scenarios [15, 16, 17, 18, 19, 20, 21, 22, 23, 24, 25, 26, 27, 28, 29]. Ultimately, AI can generate a proposed implant plan tailored to the patient's specific anatomy, based on comprehensive virtual patient modeling created through CBCT–IOS segmentation and registration [30, 31, 32, 33, 34]. AI has demonstrated high accuracy in maxillofacial anatomical segmentations ranging from 92% to 99.7%, precise IOS to CBCT registration with a point‐based deviation of 0.21 mm and a surface‐based deviation of 0.22 mm [18, 19, 20, 21].

AI has demonstrated significant potential across multiple stages of implant planning workflow, including the assessment of bone quality and density, automatic segmentation of anatomical structures, and identification of critical landmarks, such as nerves and sinus cavities [7]. AI‐driven decision‐support systems can recommend optimal implant positions, angulations, and drilling protocols based on patient‐specific bone characteristics, thereby improving precision and reducing operator variability. Furthermore, the integration of AI with augmented and virtual reality technologies has enhanced both surgical visualization and patient communication, allowing real‐time simulation of implant placement scenarios [14]. Recent studies have also shown that AI‐assisted virtual implant planning can achieve up to 93% clinical acceptability compared with expert human planning, underscoring its feasibility and growing clinical relevance in digital implant dentistry [30, 31, 32, 33, 34, 35].

To the best of our knowledge, there is however a gap in the literature regarding the clinical applicability of AI in virtual implant placement. Previous studies have largely focused on predicting implant location or comparing AI‐driven planning with human‐based virtual planning, without validating AI outcomes against actual clinical placements [30, 31, 32, 33, 34]. The present study would attempt to bridge the gap with the clinical applications by extending the evaluation to the actual implant placement. More specifically, it would compare AI‐driven and human expert virtual placement with actual implants surgically placed in the posterior mandible. Therefore, the objective of this study was to assess the performance of a validated AI‐based platform for virtual implant placement, comparing it to human expert planning and actual clinical outcomes in terms of implant location, dimensional selection, time efficiency, and consistency for single implants placed in the posterior mandible.

## 2 Material and Methods

This retrospective study was approved by the Local Ethics Committee at the University Hospitals Leuven (UZ Leuven), Belgium (reference number: S66447), and was conducted in accordance with Good Clinical Practice (ICH–GCP) guidelines, the most recent revision of the Declaration of Helsinki, and the Oviedo Convention on Human Rights and Biomedicine [36]. To safeguard patient confidentiality, all patient data were anonymized and informed consent was waived by the Ethics Committee.

### 2.1 Dataset

A dataset of 32 patient cases who underwent implant placement for a single missing mandibular premolar or molar at UZ Leuven, Belgium, was selected for this study, consisting of 21 males and 11 females, with a mean age of 55 ± 12 years. Each case included at least one preoperative CBCT scan (i.e., before implant placement), one postoperative CBCT scan (i.e., after implant placement, obtained as part of the implant follow‐up or for other clinical reasons such as new implant placement or case‐specific needs, as requested by the surgeon), and one IOS acquired prior to implant placement. CBCT scans were acquired using the NewTom VGI Evo device (Cefla, Imola, Italy), while IOS data were acquired with the Trios 3 scanner (3Shape, Copenhagen, Denmark).

Cases were included based on specific criteria that required the presence of a single missing mandibular premolar or molar within a bounded edentulous space or free‐end saddle (i.e., at least one adjacent anterior tooth). Additionally, the postoperative CBCT scan had to be obtained within 6 months of the preoperative scan. Cases that required soft‐tissue or bone augmentation during implant placement were excluded, as well as those with motion or severe beam‐hardening artifacts, missing IOS, or incomplete or unclear preoperative or postoperative CBCT data. Out of the 32 included cases, 23 involved missing molars and 9 missing premolars, with 22 single bounded missing (pre)molars and 10 free‐end saddle missing (pre)molars.

### 2.2 Data Handling

**FIGURE 1:**
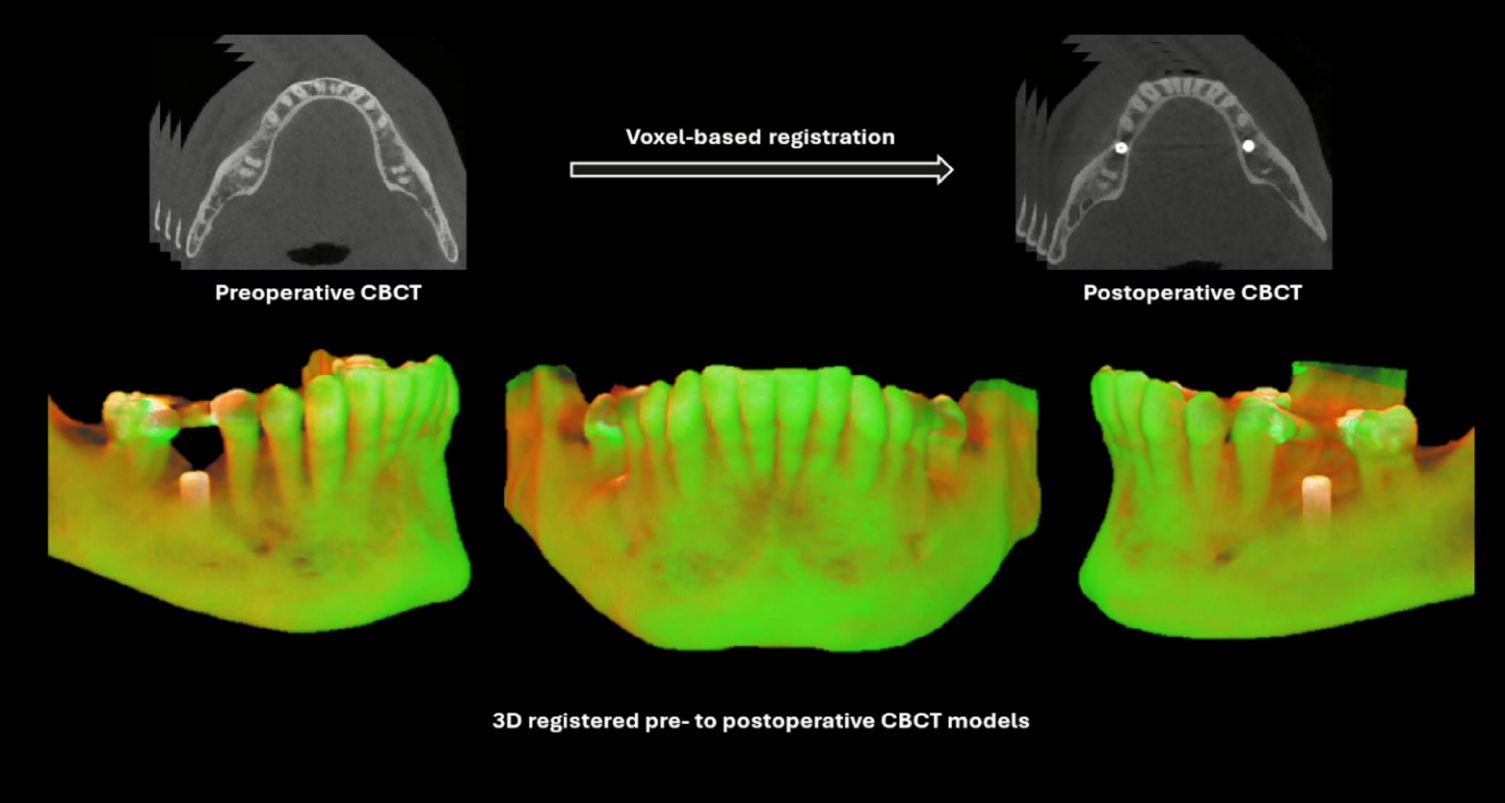
Registration of preoperative and postoperative CBCT scans in Amira software. Green: preoperative CBCT model; yellow: postoperative CBCT model.

All preoperative and postoperative CBCT scans were exported as Digital Imaging and Communications in Medicine (DICOM) files, while IOS were exported as Standard Tessellation Language (STL) files. For each case, the preoperative CBCT scan was registered to the postoperative CBCT scan using Amira software (Thermo Fisher Scientific, Mérignac, France) done with voxel‐based registration (Figure 1). The registration process was conducted by an experienced operator (E.A.D.), with over 10 years of experience in dentistry, and subsequently verified by an oral and maxillofacial radiology specialist (R.C.F.). Upon completion of registration, the registered preoperative CBCT scans were exported as DICOM files for further analysis.

Subsequently, both preoperative and postoperative CBCT scans were uploaded by the same operator to the virtual patient creator cloud‐based platform (Relu creator, Leuven, Belgium). Within this platform, a virtual patient (3D segmentation of teeth, implant restoration, bone, and mandibular canal structures) was automatically generated by AI for each case (one for preoperative and one for postoperative). Additionally, the preoperative virtual patient included the IOS, which was automatically registered by the platform into the CBCT model. This integration provided a comprehensive overview of the soft tissues, enhancing visualization for subsequent implant planning. The accuracy of anatomical landmarks segmentation and subsequent IOS registration in this AI‐driven process has been validated in previous studies [15, 16, 17, 18, 19, 20, 21, 22, 23, 24, 25, 26, 27, 28, 29].

### 2.3 Actual and Virtual Implant Placement

In this study, three implant placements were compared for each case:

1. *Human intelligence‐driven implant placement (HI‐1)*: Implant dentistry specialists, including maxillofacial surgeons and periodontists, carried out the clinical implant placements at UZ Leuven Hospital in Leuven, Belgium. Preoperative CBCT scans were used for planning, and all implants were placed freehand. As this was a retrospective study, all implant placements had been completed prior to the initiation of the study and were not influenced by the study protocol. The implant positions were determined solely by the clinical judgment of the treating specialists. The specialists also selected a variety of implant brands at their discretion. The Astra Tech EV implant (Dentsply Sirona, Mölndal, Sweden) was the most used implant, used in 13 cases. Straumann implants (Straumann AG, Basel, Switzerland) were used in eight cases. Nobel Replace and Nobel Parallel implants (Nobel Biocare, Zurich, Switzerland) were used in seven and two cases, respectively. Additionally, Astra Osseospeed implants (Dentsply Sirona, Mölndal, Sweden) were used in two cases.
2. *Artificial intelligence‐driven implant planning (AI)*: Using the same preoperative virtual patient in Relu platform (Relu creator, Leuven, Belgium), the operator initiated the implant planning process by selecting the desired tooth position. The AI then generated a proposed implant (Biotech, Salon‐de‐Provence, France) plan within seconds. This platform has previously been validated for virtual implant planning in earlier studies. The AI model employed supervised multiple 3D U‐networks for both coarse and fine anatomical landmark segmentation. For CBCT–IOS registration, 3D U‐networks were applied for the initial alignment, followed by refinement using an AI‐based iterative closest point (ICP) algorithm. Implant placement was guided by machine learning restriction values informed by prior knowledge of adjacent AI‐segmented 3D anatomical structures (i.e., neighboring teeth, mandibular canal, and buccal and lingual cortical plates) [30, 31]. The AI was trained to determine the optimal implant location and dimension by considering anatomical constraints, including maintaining a minimum distance of 2 mm from the mandibular canal, 1.5 mm from adjacent teeth, and 3 mm from adjacent implants. Additionally, the AI aimed to achieve a compromise in the implant long axis by balancing between the automatically generated wax‐up long axis and the long axis of the adjacent teeth.
3. *Human intelligence‐driven implant planning (HI‐2)*: Within the same platform, virtual implant placement was manually done for all cases by an expert in implant prosthodontics (B.M.E.), who determined the optimal implant location and dimension based on clinical experience, the available alveolar bone, and a wax‐up prepared to represent the prosthetic space. Each plan was subsequently reviewed by an expert in periodontology (P.L.). Any discrepancies between the two specialists were resolved through discussion, and when consensus could not be achieved, a third senior implant dentistry expert with more than 20 years of experience (R.J.) was consulted. This consensus‐based approach ensured that each finalized plan represented a clinically reliable reference for subsequent analysis.

**FIGURE 2:**
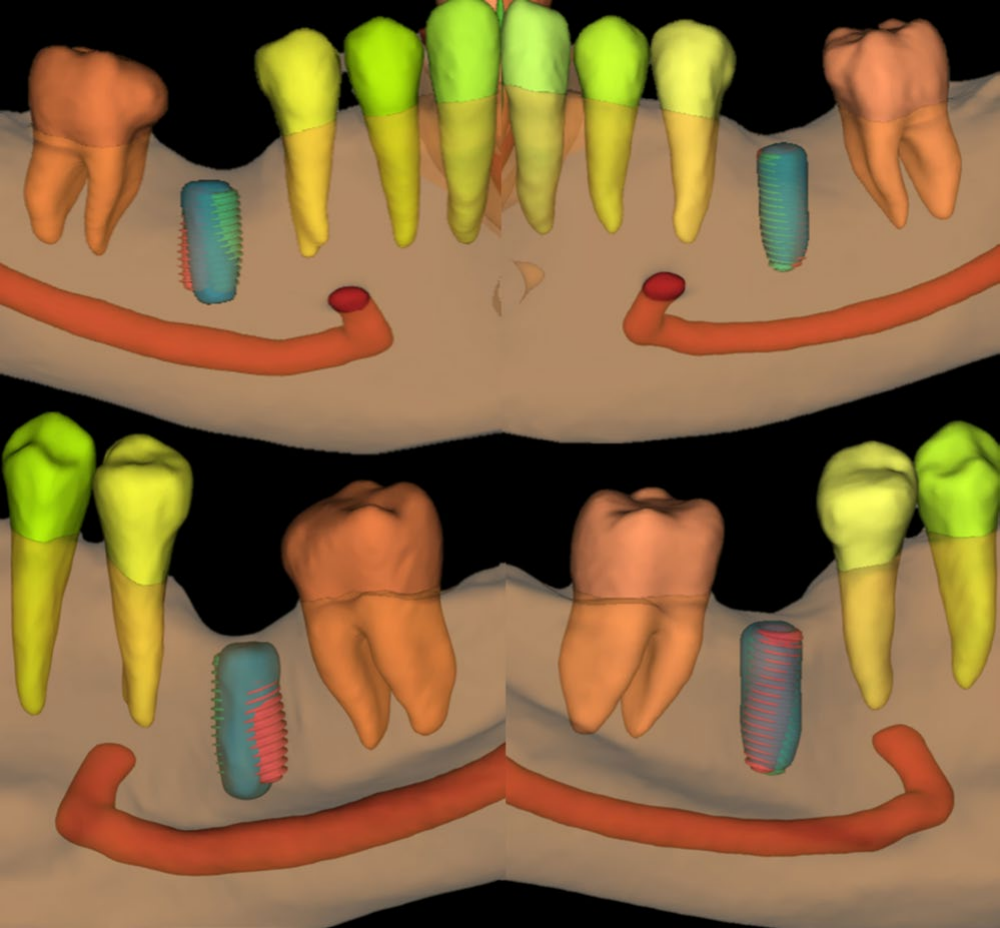
AI‐driven virtual planning of bilateral missing mandibular first molars, showing three implants: human intelligence‐placed implant (HI‐1, blue), AI‐planned implant (AI, red), and human intelligence‐planned implant (HI‐2, green), together with relevant anatomical landmarks (mandibular jawbone, teeth, and mandibular canal).

Consequently, for each case, three implants were exported as STL files: one representing the actual implant placed in the patient's mouth (HI‐1) and two from the virtual planning process, one by AI‐planned (AI) and one planned by experts (HI‐2). The actual placed implant was segmented using the previously mentioned platform; this segmentation process was fully automated by AI and its accuracy in implant segmentation has been validated in previous studies [17, 37]. Figure 2 illustrates the three implants within the virtual patient generated from the registered preoperative CBCT scans.

### 2.4 Implant Location Assessment

**FIGURE 3:**
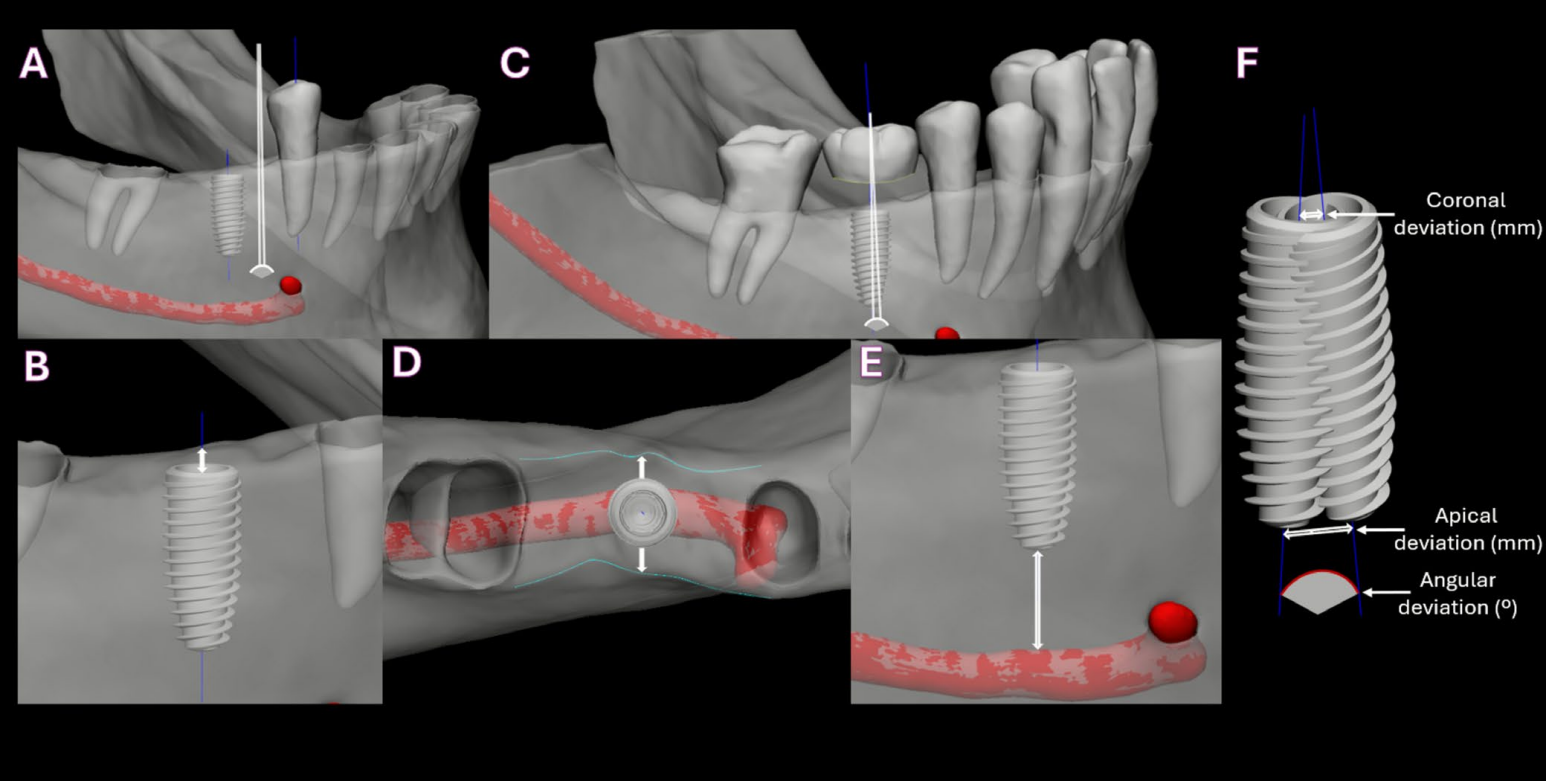
Quantitative criteria for comparing implant placement/planning in relation to surrounding anatomical structures and designed wax‐up. (A) Angle between the implant's long axis and the adjacent anterior tooth's long axis; (B) crestal bone height between the implant platform and the crestal bone surface; (C) angle between the implant's long axis and the long axis of the wax‐up; (D) buccal and lingual bone thickness around the implant; (E) distance between the implant apex and the mandibular canal; (F) comparative relation between two planned implants (AI‐planned and human intelligence‐planned [HI‐2]), focusing on coronal and apical deviations as well as the angular deviation between their long axes.

For each case, a wax‐up design for the missing tooth was created on the Relu platform by an expert in prosthetic dentistry (E.A.D.) using the registered cast generated from the alignment between the CBCT and IOS scans. A quantitative comparison was performed among the HI‐1, AI, and HI‐2 implants. All three implant STL models, along with the AI‐segmented mandible, mandibular teeth, mandibular canal (from the preoperative virtual patient), and the expert‐designed wax‐up, were imported into the 3‐matic software (Materialise, Leuven, Belgium) for detailed analysis. This assessment included the spatial relationship of each implant to adjacent anatomical structures and the designed wax‐up, providing a comprehensive comparison of the three planning approaches. Figure 3 illustrates the evaluation criteria used for implant location assessment.

#### 2.4.1 Relation to Adjacent Neighboring Structures

The implants planned/placed by HI‐1, AI, and HI‐2 were compared based on the following criteria:

1. *Angular relationship to adjacent anterior tooth*: The angle between the implant's long axis and the long axis of the adjacent anterior tooth (measured in degrees).
2. *Crestal bone height at the implant platform*: The height of the bone between the implant platform and the crestal bone surface (measured in millimeters)
3. *Angular alignment with wax‐up*: The angle between the implant's long axis and the long axis of the expert‐designed wax‐up (measured in degrees).
4. *Buccal and lingual bone thickness*: The thickness of the buccal and lingual bone surrounding the implant (measured in millimeters)
5. *Distance from implant apex to mandibular canal*: The distance between the implant apex and the mandibular canal (measured in millimeters).

#### 2.4.2 Relation Among Implants

This assessment was conducted based on the following criteria:

1. *Coronal deviation*: The distance, in millimeters, between the intersection of the long axis of each implant and the implant platform surface compared to the same point on the other implant.
2. *Apical deviation*: The distance, in millimeters, between the intersection of each implant's long axis and the implant apex, compared to the same point on the other implant
3. *Angular deviation*: The angle between the long axis of each implant and the long axis of the compared implant.

### 2.5 Implant Dimension Comparison

The selection of implant dimension (i.e., diameter and length measured in millimeters) was evaluated among the choices selections made by the HI‐1, AI, and HI‐2 for each missing tooth. This comparison aimed to assess the degree of difference between the AI‐driven selections and those made by human operators in determining the most appropriate implant dimension.

### 2.6 Time Efficiency

Twenty percent of the cases were randomly selected to measure the time required by the AI platform to generate implant plans, allowing for direct comparison with human planning performed by the same operator (B.M.E.). For benchmarking time efficiency, a traditional implant planning software (DTX Studio Implant version 3.6.6.1, Nobel Biocare, Kloten, Switzerland) was used for expert planning of these cases. For both approaches, the time needed from uploading the CBCT and IOS data to exporting the planned implant simulation was measured using an integrated stopwatch system.

### 2.7 Consistency

The same randomly selected 20% subset of the dataset selected to perform the aforementioned analysis was also used to evaluate the consistency between the AI and HI‐driven approaches. For each approach, implant planning was performed twice, and the consistency between the two plans was evaluated by measuring the median surface deviation (MSD) and the root mean square (RMS) deviation between the two planned implant positions. MSD represents the average distance between corresponding points on the two compared implants, while RMS reflects the degree of misalignment between them. A zero value for both metrics indicates that the two planned implants are identical in location

### 2.8 Statistical Analysis

Data analysis and sample size calculation were performed using MedCalc Statistical Software (version 20.116; MedCalc Software Ltd., Ostend, Belgium). Sample size calculation was conducted based on the findings of Elgarba et al. [31], which compared AI‐ and HI‐based implant planning. The calculation parameters were set at a significance level (*α*) of 0.05 and a power (1 − *β*) of 0.80, assuming a medium effect size (Cohen's *d* = 0.5). Using a Friedman test and considering a mean difference of 0.1 mm with a standard deviation of 0.13 mm in coronal implant deviation, the required sample size was estimated. Under these assumptions, a total of 32 participants was determined to be sufficient to detect a statistically significant difference between AI‐ and HI‐guided implant planning. Descriptive statistics were used to summarize quantitative data, with normally distributed variables presented as mean ± standard deviation (SD), while nonnormally distributed variables were reported as median ± interquartile range (IQR). Normality of data distribution was assessed using the Shapiro–Wilk test. For normally distributed data, repeated measures analysis of variance (ANOVA) followed by Tukey's post hoc test was used to evaluate implant dimension selection. For nonnormally distributed data, the Friedman test followed by Wilcoxon signed‐rank post hoc analysis was used to evaluate implant location. Additionally, a paired *t*‐test was conducted to analyze the differences between the AI and HI approaches in terms of time efficiency and consistency. A *p*‐value of less than 0.05 was considered statistically significant for all analyses.

## 3 Results

### 3.1 Implant Location Performance

#### 3.1.1 Relation to Adjacent Neighboring Structures

Table 1 presents the comparative analysis of implant positioning performed by HI‐1, AI, and HI‐2 based on their proximity to adjacent anatomical landmarks and alignment with the designed wax‐up. The results were stratified by bounded and free‐end cases, as well as presented for the entire dataset. A statistically significant difference (*p* < 0.05) among the three methods was observed when analyzing the entire dataset in terms of crestal bone height and angular alignment with the designed wax‐up. For crestal bone height, HI‐2 showed comparable results (1.1 ± 0.5 mm) to both AI and HI‐1 (1.1 ± 0.6 mm and 0.8 ± 0.8 mm, respectively), although AI and HI‐1 differed significantly from each other (*p* = 0.01). Conversely, for alignment with the wax‐up design, AI and HI‐1 exhibited similar results (5.5° ± 4.2° and 5.5° ± 3.4°, respectively), while HI‐2 differed significantly from both (4.3° ± 2°) (*p* = 0.01). Furthermore, no significant differences were observed (*p* > 0.05) across all other comparison metrics among HI‐1, AI, and HI‐2 for the entire dataset.

**TABLE 1:** Relation of artificial intelligence (AI)‐planned, human intelligence (HI)‐placed/planned implants with reference landmarks between bounded and free‐end cases, as well as across the entire dataset.

Metric	Median ± IQR									*p*‐Value (entire dataset comparison)
	Bounded (*n* = 22)			Free‐end (*n* = 10)			Entire dataset (*n* = 32)			
	HI‐placed (HI‐1)	AI‐planned (AI)	HI‐planned (HI‐2)	HI‐placed (HI‐1)	AI‐planned (AI)	HI‐planned (HI‐2)	HI‐placed (HI‐1)	AI‐planned (AI)	HI‐planned (HI‐2)	
Angle with anterior adjacent tooth (°)	7.1 ± 6.5	7.1 ± 6	6.4 ± 4.4	9 ± 4	5.2 ± 3	3.6 ± 6.7	7.7 ± 5.6	6 ± 4.7	5.2 ± 5.7	0.1
Crestal bone height (mm)	0.8 ± 0.6	1.1 ± 0.4	1 ± 0.3	1.3 ± 1.6	1.3 ± 0.8	1.2 ± 0.8	0.8 ± 0.8A	1.1 ± 0.6B	1.1 ± 0.5AB	0.01*
Angle with wax‐up (°)	5.2 ± 3	6.5 ± 5	4.3 ± 2.1	9.2 ± 8.5	4.7 ± 2.4	4 ± 2	5.5 ± 3.4A	5.5 ± 4.2A	4.3 ± 2B	0.01*
Buccal bone thickness (mm)	2 ± 2.3	1.8 ± 3	1.8 ± 2.8	3.1 ± 1.2	2.5 ± 2.3	2.4 ± 1.9	2.4 ± 2	2.2 ± 2.7	2 ± 2.5	0.83
Lingual bone thickness (mm)	1.9 ± 1.3	1.7 ± 1.1	1.7 ± 1.3	1.7 ± 0.9	1.8 ± 1.5	1.8 ± 1.6	1.8 ± 1.2	1.7 ± 1.2	1.7 ± 1.4	0.44
Distance to mandibular canal (mm)	5.2 ± 2.6	6.2 ± 2	5.6 ± 2.1	5 ± 2.8	4.1 ± 3.4	4 ± 3	5.2 ± 2.9	6.1 ± 2.7	5.5 ± 2.8	0.69

*Note:* An asterisk (*) indicates statistical significance (*p* < 0.05) among the implant placement approaches for the entire dataset using Friedman test; different uppercase letters (e.g., A and B) indicate a statistically significant difference as determined by the Wilcoxon signed‐rank post hoc test.

Abbreviations: ° = degree; AI = artificial intelligence; HI‐1 = human intelligence‐placed implants; HI‐2 = human intelligence‐planned implants; IOR = interquartile range; mm = millimeter.

#### 3.1.2 Relation Among Placed Implants

Table 2 presents the results of the quantitative comparison among the three implants (HI‐1, AI, and HI‐2) for each case. For the entire dataset, a significant difference was observed in both apical deviation and angular deviation among the implants (*p* < 0.05), whereas coronal deviation did not show a significant difference (*p* > 0.05). Notably, coronal deviation remained within a clinically acceptable range, remaining below 1 mm. Although the apical deviation between AI and HI‐2 was also less than 1 mm, both AI and HI‐2 exhibited greater apical deviation when compared with HI‐1.

**TABLE 2:** Relation among artificial intelligence (AI)‐planned, human intelligence (HI)‐placed/planned implants within bounded and free‐end cases, as well as across the entire dataset.

Metric	Bounded (*n* = 22)			Free‐end (*n* = 10)			Entire dataset (*n* = 32)				Post hoc *p*‐values (entire dataset comparison)		
	AI vs. HI‐1	AI vs. HI‐2	HI‐1 vs. HI‐2	AI vs. HI‐1	AI vs. HI‐2	HI‐1 vs. HI‐2	AI vs. HI‐1	AI vs. HI‐2	HI‐1 vs. HI‐2	*p*‐Value (entire dataset comparison)	AI vs. HI‐1 and AI vs. HI‐2	AI vs. HI‐1 and HI‐1 vs. HI‐2	AI vs. HI‐2 and HI‐1 vs. HI‐2
	Median ± IQR												
Coronal deviation (mm)	0.9 ± 0.6	0.8 ± 0.3	1 ± 0.7	1 ± 1.5	0.9 ± 0.4	1.3 ± 1.5	0.9 ± 0.8	0.8 ± 0.4	1 ± 1.1	0.09	N/A	N/A	N/A
Apical deviation (mm)	2 ± 1.1	1 ± 1	1.8 ± 0.9	2 ± 0.6	0.6 ± 1	1.7 ± 0.8	2 ± 1	0.9 ± 1	1.8 ± 1	0.001*	0.001*	0.5	< 0.001*
Angular deviation (°)	6.2 ± 6.4	5.1 ± 4.2	5.3 ± 3.9	9.1 ± 5.5	3.3 ± 3.2	8.8 ± 3.7	7.6 ± 6.6	4.5 ± 4.1	6.6 ± 4.2	0.03*	0.002*	0.18	0.013*

*Note:* An asterisk (*) denotes statistical significance (*p* < 0.05) for the entire dataset, indicating overall differences among implant placement approaches as determined by the Friedman test and for each pairwise comparison between two individual means, as assessed by the Wilcoxon signed‐rank post hoc test.

Abbreviations: ° = degree; AI = artificial intelligence‐planned implants; HI‐1 = human intelligence‐placed implants; HI‐2 = human intelligence‐planned implants; IOR = interquartile range; mm = millimeter; N/A = not applicable.

### 3.2 Implant Dimension Selection

Table 3 presents the comparison of implant diameter and length selection among HI‐1, AI, and HI‐2 approaches. No significant differences were observed in the selection of implant dimension (*p* > 0.05), indicating a high similarity among the three approaches in determining implant size.

**TABLE 3:** Comparison among artificial intelligence (AI)‐based planning, human intelligence (HI)‐based placement and planning in selecting implant diameter and length across the entire dataset.

Metric	Mean ± SD			
	HI‐placed (HI‐1)	AI‐planned (AI)	HI‐planned (HI‐2)	*p*
Implant diameter (mm)	4.3 ± 0.3	4.3 ± 0.4	4.3 ± 0.4	0.47
Implant length (mm)	9.7 ± 1.3	9.9 ± 1.2	9.8 ± 1.3	0.19

*Note:* No significant difference was found in diameter and length selection among HI‐1, AI, and HI‐2 as determined repeated measure ANOVA (*p* > 0.05).

Abbreviations: HI‐1 = human intelligence‐placed implants; AI = artificial intelligence; HI‐2 = human intelligence‐planned implants; mm = millimeter; SD = standard deviation.

### 3.3 Time Consumption

A significant difference was observed between the AI‐ and expert‐based implant planning approaches in the time taken for the overall implant planning process, from CBCT and IOS data upload to planned implant export (*p* = 0.0002). The AI approach demonstrated a more than tenfold increase in time efficiency compared to the HI‐2 method, with a mean processing time of 36.3 ± 7.3 s versus 373 ± 113 s, respectively.

### 3.4 Consistency

A significant difference was observed between AI‐ and HI‐based implant planning approaches in the consistency of the virtual implant placement workflow, considering both MDS (*p* = 0.004) and RMS (*p* = 0.002) metrics. The AI approach demonstrated excellent consistency with zero‐degree surface deviation (MSD = 0.00 ± 0.00 mm and RMS = 0.00 ± 0.00 mm), whereas the HI method exhibited deviations of 0.39 ± 0.23 mm for MSD and 0.68 ± 0.23 mm for RMS.

## 4 Discussion

Key factors for long‐term implant success include meticulous planning of the ideal implant position and dimension, as this directly influences both functional and esthetic outcomes [38, 39]. Optimal implant placement ensures appropriate load distribution, preserves the integrity of the surrounding bone, and facilitates prosthetically driven implant placement, simplifying the restorative phase and reducing the risk of biomechanical complications [40, 41]. A comprehensive assessment of multiple clinical parameters (e.g., bone volume and quality, anatomical landmarks, position of adjacent and opposing teeth, and soft tissues status) during the planning phase is essential to ensure predictable and successful outcomes [42]. Advanced technologies, including digital planning tools, enhance the understanding and predictability of each clinical situation by enabling customized treatment plans while AI integration aims to offer significant advantages such as reduced surgery time, high consistency, and accuracy comparable to that of experienced clinicians [7, 43, 44].

Despite the increasing availability of AI‐based solutions in the dental market, clinical validation remains limited in the current literature [7, 44]. This study's novelty lies in its comprehensive evaluation of AI‐driven virtual implant placement. It compares this method not only to human‐operated virtual planning, but also to implants actually placed in patients by implant dentistry specialists. This dual‐level comparison provides a more clinically relevant assessment of AI performance, as it reflects the realities of implant placement where different specialists, with varying levels of expertise and clinical backgrounds, may arrive at different planning and placement decisions. As such, the study offers a more clinically engaging perspective that mirrors daily implant workflows. The findings demonstrated that AI achieved clinically acceptable implant positioning in cases involving missing single mandibular premolars and molars, regardless of whether the edentulous space was bounded or a free‐end saddle. Furthermore, AI outperformed human operators in time efficiency, being approximately 10 times faster while maintaining a high level of consistency across all planned cases.

The performance of both virtually and actually placed implants was evaluated by assessing their relationship to adjacent anatomical structures and the designed wax‐up, which served as consistent reference points across all placement approaches [45]. This methodology enabled a precise comparison among the different implant placement modalities. These findings indicate that preoperative planning, whether performed by human operators or AI, tended to result in implants being planned slightly deeper than those actually placed. For instance, the planned implants (AI and HI‐2) exhibited bone thickness above the implant of 1.1 ± 0.6 mm and 1.1 ± 0.5 mm, respectively, whereas the actual placed implants showed significantly less bone thickness (0.8 ± 0.8 mm). This discrepancy may be explained by the practical limitations during surgery, where implants cannot always be positioned as deep as planned, or it may reflect expected bone resorption around the implant following placement [46].

Furthermore, dental implant placement fundamentally requires a careful balance between the available alveolar bone and the desired prosthetic outcome, typically guided by a diagnostic wax‐up during the planning process [47]. In this study, AI demonstrated clinically acceptable buccal and lingual bone thicknesses and angulation relative to the designed wax‐up, with values of 2.2 ± 2.7 mm, 1.7 ± 1.2 mm, and 5.5° ± 4.2°, respectively. The HI‐2 group showed comparable buccal and lingual bone thicknesses (2 ± 2.5 mm and 1.7 ± 1.4 mm) and a slightly more favorable angulation of 4.3° ± 2°. The HI‐1 group achieved buccal and lingual bone thicknesses of 2.4 ± 2 mm and 1.8 ± 1.2 mm, with an angulation of 5.5° ± 3.4° relative to the wax‐up. These findings suggest that AI can maintain an effective balance between anatomical constraints and prosthetic requirements when selecting implant positions, supporting its reliability in prosthetically driven planning.

When evaluating virtually and actually placed implants, the coronal deviation between each pair remained within approximately 1 mm, regardless of the planning approach used. This indicates that despite the inherent differences in planning approach among HI‐1, AI, and HI‐2, all methods converged on an almost identical emergence profile for the implant. In contrast, larger apical deviations were noted, likely reflecting discrepancies in the planned implant angulation and variations in the selection of implant lengths between the different approaches. These findings are consistent with those reported by Elgarba et al. [31], Satapathy et al. [32], and Cai et al. [48], reinforcing the idea that while the entry point of implant placement remains consistent, the angulation may vary depending on the planning strategy employed.

In the present study, AI‐based planning resulted in a mean coronal deviation of 0.9 ± 0.8 mm compared with HI‐placed implants and 0.8 ± 0.4 mm compared with HI‐planned implants. The mean apical deviation was 2.0 ± 1.0 mm compared to the HI‐placed implants and 0.9 ± 1.0 mm compared to the HI‐planned implants. Additionally, the angular deviation was 7.6° ± 6.6° relative to the placed implants and 4.5° ± 4.1° relative to the planned implants. These findings indicate a high level of consistency between AI‐ and HI‐guided implant planning and placement. The magnitude of observed deviation aligns closely with the clinically acceptable thresholds for implant positioning accuracy reported in previous literature [47, 48, 49]. This consistency further supports the feasibility and potential clinical applicability of AI‐assisted planning systems in achieving a comparable level of precision as human experts.

Regarding the angulation between each pair of placed implants, the comparison between AI and HI‐2 revealed a smaller angular deviation (4.5° ± 4.1°) than those involving HI‐1 (7.6° ± 6.6° for AI vs. HI‐1 and 6.6° ± 4.2° for HI‐1 vs. HI‐2). These findings further support the notion that freehanded surgical implant placement is associated with a higher risk of directional misalignment in the absence of guided surgery. The observed deviations are in line with previously published studies, such as those by Tan et al. [47], who reported deviations ranging from 3.9° ± 2.9° to 8.8° ± 5°, and Tang et al. [49], who found a mean angular deviation of 7.9° ± 5.6°, highlighting the discrepancies between preoperative planning and final implant position in clinical practice [47, 49].

The choice of implant dimension by HI‐1, AI, and HI‐2 revealed a high degree of similarity, as no difference was observed among the approaches studied for both diameter and length selection. These findings are consistent with those reported by Elgarba et al., where the AI selected implants had a mean diameter of 4.0 ± 0.3 mm and a mean length of 11.7 ± 1.3 mm, compared to 4.2 ± 0.4 mm and 11.5 ± 1.3 mm, respectively, for HI [31]. It should be noted that slight variations in implant diameter and length across the different planning approaches investigated may be attributed to the use of different implant systems or brands. For the implants placed by specialists, the choice of implant brand was primarily driven by individual clinical preference. Furthermore, the lack of standardization of implant brands in cases with previously placed implants (i.e., each brand offers implants with different dimension) probably contributed to the observed differences.

One of the major advantages of integrating AI into implant planning workflow is its exceptional time efficiency and high consistency. In this study, AI completed the planning process 10 times faster than human operators while maintaining perfect reproducibility. In comparison, HI‐planned implants demonstrated lower consistency based on the higher MSD and RMS values achieved. These findings highlight the potential of AI as a time‐saving and reliable clinical assistant, offering consistent performance unaffected by human‐related factors such as fatigue or emotional state. These results are consistent with previous studies validating AI integration into various digital dental workflows, including anatomical landmark segmentation, IOS to CBCT registration, and preoperative planning [7, 13, 19, 26, 28, 29, 37, 50, 51].

This study presents a novel clinical validation of AI‐driven virtual implant placement for missing mandibular (pre)molar implant rehabilitation cases, addressing a significant gap in the current literature, as most AI‐based digital workflows lack robust clinical validation. Uniquely, AI performance was evaluated not only against human‐driven planning but also against implants actually placed by specialists from different disciplines (i.e., maxillofacial surgery and periodontology), providing a realistic representation of routine clinical practice. However, one potential limitation of this study is the unavailability of the original planning files from the respective specialists. In most cases, the implant planning was not performed by the same clinician who conducted the surgical placement; instead, it was prepared by different staff members and subsequently used only as a reference during the procedure. This prevented a direct comparison between the specialists' initial plans, the AI‐generated plans, and the final clinically placed implants. To overcome this limitation, two expert clinicians (i.e., one in periodontology and one in implant prosthodontics) replanned all cases from the beginning, ensuring a standardized and reliable planning process. Nevertheless, future prospective studies are recommended to further validate the use of AI for virtual implant placement by directly comparing AI‐generated outputs with both the planning and the clinical outcomes of specialized practitioners who perform both the planning and the actual implant placement.

Furthermore, AI model development and validation typically follow a curriculum‐based testing approach, beginning with single‐center datasets and gradually progressing to multicenter studies. Future research would greatly benefit from incorporating more diverse datasets from different sources, as this would provide a more realistic and generalizable environment for validating AI tools in dental implant planning. Likewise, the validated AI model should be tested across a variety of clinical scenarios beyond missing posterior mandibular teeth, since each clinical situation has its own specific requirements determined by anatomical limitations as well as functional and esthetic demands. Furthermore, as multiple outcomes were tested without adjustment for multiplicity, the possibility of type I error cannot be excluded. In particular, results with borderline *p*‐values should be interpreted with caution. Future studies with larger sample sizes and prespecified primary endpoints, along with appropriate statistical adjustments for multiplicity, are needed to confirm these exploratory findings.

## 5 Conclusion

The AI‐driven platform automatically selected both implant location and dimension for missing mandibular premolars and molars, yielding results that were well within clinically acceptable limits when compared with both human expert plans and the actual placements performed by implant specialists. Notably, the AI‐driven platform demonstrated superior time efficiency and greater consistency compared to human operators. These findings suggest that AI holds considerable potential for clinical integration into virtual implant planning workflows. Nevertheless, further validation studies are needed to confirm the applicability of AI models across different clinical scenarios. Future research should also compare the outcomes of these models with those of alternative software platforms and human operators.

## Author Contributions

**Bahaaeldeen M. Elgarba:** conceptualization, methodology, resources, data curation, visualization, investigation, writing – original draft, writing – review and editing. **Rocharles Cavalcante Fontenele:** formal analysis, visualization, investigation, writing – review and editing. **Eslam Abdelwahab Dawood:** methodology, validation. **Pierre Lahoud:** methodology, validation. **Jan Meeus:** validation, resources. **Reinhilde Jacobs:** conceptualization, methodology, supervision, writing – review and editing.

## Funding

This work was supported by the researcher (Bahaaeldeen M. Elgarba) is funded by a full scholarship (GM01‐2020) from the Ministry of Higher Education of the Arab Republic of Egypt.

## Conflicts of Interest

The authors declare no conflicts of interest.

## Data Availability

The data that support the findings of this study are available from the corresponding author upon reasonable request.
